# SEOM—GECOD clinical guideline for unknown primary cancer (2021)

**DOI:** 10.1007/s12094-022-02806-x

**Published:** 2022-03-23

**Authors:** Ferrán Losa, Isaura Fernández, Olatz Etxaniz, Alejandra Giménez, Paula Gomila, Lara Iglesias, Federico Longo, Esteban Nogales, Antonio Sánchez, Gemma Soler

**Affiliations:** 1 Hospital de Sant Joan Despí Moisés Broggi-ICO Hospitalet, Barcelona, Spain; 2 Hospital Álvaro Cunqueiro-IIGS, Vigo, Spain; 3 Hospital Germans Trias I Pujol -ICO Badalona, Barcelona, Spain; 4 Hospital Lluís Alcanys de Xàtiva, Valencia, Spain; 5 Hospital Miguel Servet (Zaragoza)/H, de Barbastro, Spain; 6 Hospital Universitario 12 de Octubre, Madrid, Spain; 7 Hospital Universitario Ramón y Cajal, IRYCIS, CIBERONC, Madrid, Spain; 8 Hospital Universitario Virgen Macarena, Sevilla, Spain; 9 Hospital Universitario Puerta de Hierro Majadahonda, Madrid, Spain; 10 Hospital Durán i Reynals-ICO Hospitalet, Barcelona, Spain

**Keywords:** Cancer, Unknown primary site, Diagnosis, Treatment

## Abstract

Cancer of unknown primary site (CUP) is defined as a heterogeneous group of tumors that appear as metastases, and of which standard diagnostic work-up fails to identify the origin. It is considered a separate entity with a specific biology, and nowadays molecular characteristics and the determination of actionable mutations may be important in a significant group of patients. In this guide, we summarize the diagnostic, therapeutic, and possible new developments in molecular medicine that may help us in the management of this unique disease entity.

## Introduction

Cancer of unknown origin (CUP) represents a heterogeneous group of tumors with a histologically confirmed diagnosis of cancer, which originates as metastatic disease, and of which, after a systematic search for the primary tumor, including at least clinical history, physical examination, blood tests and biochemistry, along with computed thoraco-abdominal tomography (CT), the origin of the primary tumor is not found.

Currently CUP represents between 3–5% of diagnosed tumors, being approximately the tenth most frequent tumor in incidence.

Given the poor prognosis of cancer of unknown origin, it is necessary to optimize diagnosis and knowledge of the potential molecular pathways involved, in order to establish therapeutic strategies, and clinical trials are currently underway to explore these pathways.

## Metodology

This guideline has been developed with the consensus of ten oncologists highly experienced in the diagnosis and treatment of CUP, and members of the Spanish Research Group on Cancer of Unknown Origin (GECOD) and the Spanish Society of Medical Oncology (SEOM).

In order to assign the levels of evidence and the grade of recommendation for the different states of this treatment guideline, The Infectious Diseases Society of America-US Public Health Service Grading System for ranking Recommendations in Clinical Guidelines have been followed [1].

## Epidemiology. biological background and proportional distribution according to occult primary site

CUP accounted for 3–5% of all diagnosed cancer in the historical series but in recent publications its incidence seems to decline. The CUP incidence is influenced by the non-existence of international consensus on the definition, classifications and registration of CUP. There is a lack of a diagnostic process record for these patients [2].

There is no gender difference, and the average age of presentation is 60. Among individuals older than 60 years of age, the incidence-rate for CUP in the digestive tract has increased markedly [2].

Little is understood about the disease pathogenesis and biology. Two predominant theories exist:

- Parallel progression model: CUP tumors are metastatic tumors that have arisen from an undetectable or regressed primary lesion. The dissemination of primary tumor cells is an early event where subsequent clonal evolution of the metastasis is distinctly different from that of the primary tumor.
- No primary tumor exists: CUP is a single metastatic entity. Metastasis occurs without parallel progression, with the tumor microenvironment selectively favoring the outgrowth of tumor cells at the metastatic site, while it avoids the growth of these genetically identical cells at the primary site [3].

The process that generates CUP is driven by multiple interdependent alterations in cell behavior, including chromosomal alterations, self-sufficiency in growth signals, resistance to growth-inhibitory signals, reprogramming of energy metabolism, evasion of apoptosis, limitless replicative potential, sustained angiogenesis, tissue invasion and metastasis and evasion from immune destruction [4].

## Prognosis. Subsets of patients. Prognostic groups

The prognosis of patients with CUP is generally poor, since by definition they are aggressive cancers that are metastatic at their onset. However, a correct diagnosis can identify a subgroup of patients around 20% with a more favorable prognosis, in whom a greater benefit can be expected when treated with a suitable treatment.

This group of patients (Table 1), called favorable prognosis, should be treated similarly to known equivalent primary tumors with metastatic spread, and may achieve long-term control of metastatic spread in 30–60% of cases [5].

**Table 1:** Favorable Prognosis Group

Adenocarcinoma with a molecular profile or IHC of colon cancer (CK20 + , CK7-, CDX2 +)
Poorly differentiated carcinoma with midline nodal distribution in men
Squamous cell carcinoma with head and neck lymph node involvement
Papillary adenocarcinoma of the peritoneal cavity in women
Adenocarcinoma involving only axillary lymph nodes in women
Blastic bone metastases and elevated PSA in men
Neuroendocrine carcinomas of unknown primary site
Squamous carcinoma in isolated inguinal nodes
Single or potentially resectable metastases

Unfortunately, most patients with CUP do not belong to these specific subgroups. Eighty percent of all patients diagnosed with CUP have a poor response to treatment and a poor prognosis, with a median overall survival of six months [6].

The prognosis of CUP is classified primarily by performance status and serum lactate dehydrogenase (LDH) level. Those with a good performance status (0–1) and a normal LDH value have a median overall survival of 12 months. Those with one or both prognostic factors have a median overall survival of only 4 months [7].

Other factors predictive of a poor outcome are: male sex, high comorbidity, age over 64 years, smoking history of more than 10 pack-years, weight loss, lymphopenia, low serum albumin concentrations and high alkaline phosphatase concentrations [8].

## Diagnostic workup

The diagnostic process in patients with CUP seeks to identify subgroups that can benefit from a specific therapeutic procedure, avoiding prolonged, expensive diagnostic processes of scant therapeutic benefit for the patient [9]. The diagnosis workup is summarized in the Table 2.

**Table 2:** Diagnosis workup in CUP

Assessment	Patient subset
Complete clinical history and physical examination, include head and neck and rectal examination CBC, LDH, and serum markers CT thorax, abdomen, and pelvis	All patients
Serum tumor markers	
AFP, BHCG	Midline presentation
PSA	Men with adenocarcinoma and bone metastasis
CA 125	Women with peritoneal adenocarcinoma
Mammography	All women
Breast MRI	Women with axillary adenocarcinoma
PET/CT	Selected cases:
	Cervical squamous cell carcinoma
	If radical treatment is possible
Endoscopy	Sign/symptom/IHC oriented
Octreoscan and chromogranin A	Neuroendocrine tumor CUP

*CBC* Complete blood count, *LDH* Lactate dehydrogenase, *CT* Computed tomography, *MRI* magnetic resonance imaging, *PSA* Prostate‑specific antigen, *PET/CT* Positron‑emission tomography, *IHC* Immunohistochemistry, *AFP* Serum α-fetoprotein, *BHCG* human chorionic gonadotropin, *CA 125* cancer antigen 125

### Anamnesis and physical examination

The primary clinical work-up includes complete medical history with attention to toxic habits, medical and surgical history, previous diseases, or a family history of neoplasms. Physical examination must include head and neck and rectal examination, testes in males, and pelvic/gynecologic and breast examination in women [10].

### Laboratory

These consist of complete blood count, liver and kidney function tests, electrolytes (including calcium), and LDH, since they represent important prognostic factors.

- Serum tumor markers: serum tumor markers are often elevated in a non-specific manner in patients with CUP, consequently their measurement offers no diagnostic or prognostic assistance. Exceptions are determination of serum PSA in male patients with bone metastasis, so as to exclude occult metastatic prostate cancer, germ cell tumor markers (*α*FP, *β*HCG) in patients with midline disease, serum αFP in liver-dominant disease, so as to exclude hepatocellular cancer, and CA125 in women with peritoneal involvement.

### Radiological examinations and complementary tests to identify the primary in cases of CUP include

Computed tomography (CT) thoraco-abdominal-pelvic is customary since, in addition to attempting to detect the primary, it serves as an extension study and can locate lesions that can be biopsied [11].

Mammography should be performed in cases of adenocarcinoma in women.

Positron emission tomography (PET) CT remains to be fully evaluated in large-scale prospective studies. Currently, as it is more effective in detecting additional metastases rather than the hidden primary, it should be used when radical therapy is contemplated for localized CUP: cervical head/neck nodes, axillary adenopathy and single metastatic lesions [12, 13].

### Examinations to be excluded in the absence of symptoms that indicate otherwise

- Laryngoscopy: useful in cases of cervical lymph node involvement.
- Bronchoscopy: in cases of radiological findings such as hiliar or mediastinal lymph node involvement, and pulmonary symptoms.
- Gastroscopy: if abdominal symptoms or positive fecal occult blood test.
- Colonoscopy: if abdominal symptoms or positive fecal occult blood test, or biopsy with immunohistochemistry CK20 + /CK7 − /CDX2 + .
- Testicular ultrasound: if retroperitoneal or mediastinal mass.
- Gynecologic ultrasound: if pelvic or peritoneal metastases, CK7 + on the biopsy tissue.
- Breast MRI: if adenocarcinoma with negative mammography and metastasis to axillary lymph nodes.

## Histological diagnosis

Pathological assessment of malignant tissue samples is crucial for CUP diagnosis. Core biopsy is preferred to fine-needle biopsy or cytology. Further procedures such as surgical biopsies may be considered when the initial sample is inadequate to confirm the diagnosis. Firstly, the pathological evaluation must rule out some special tumors with a specific therapeutic approach (lymphoma, germ-cell tumor, melanoma, or sarcoma) [14]. Thereafter the IHC staining may classify CUP into these five morphological subgroups [15]:

- Well/moderately-differentiated adenocarcinoma (60%).
- Poorly-differentiated adenocarcinoma/undifferentiated carcinoma (20–30%).
- Squamous-cell carcinoma (5%).
- Poorly-differentiated neoplasms (5%).
- Neuroendocrine tumors (1–3%).

## Immunohistochemistry

IHC plays an essential role in the evaluation of samples of metastatic tumors. It has a relatively low cost compared with other techniques, but it also has limitations. Nowadays, it would be important to reserve tumor material for molecular studies.

Keratins, expressed in epithelial cells, have historically been useful in confirming epithelial origin in poorly differentiated malignancies, although other tumor types may also express keratins. IHC should be applied for determining most likely cell lineage, and in order to exclude high chemo-sensitive and potentially curable tumors, and/or to rule out hormone-sensitive malignancies amenable to specific therapy. Staining for chromogranin A and synaptophysin is needed to profile neuroendocrine differentiation.

Among keratin family members, CK7 and CK20 have most widely been used to predict primary site. Although these expression patterns may be useful to prioritize one site of origin over another and to direct further workup, cases that do not fit these profiles are encountered frequently. In this sense, the CK7 positive and CK20 negative immunophenotype is the most common in CUP; however, this profile is not particularly useful for suggesting a specific anatomical site of origin [16].

**Fig. 1:**
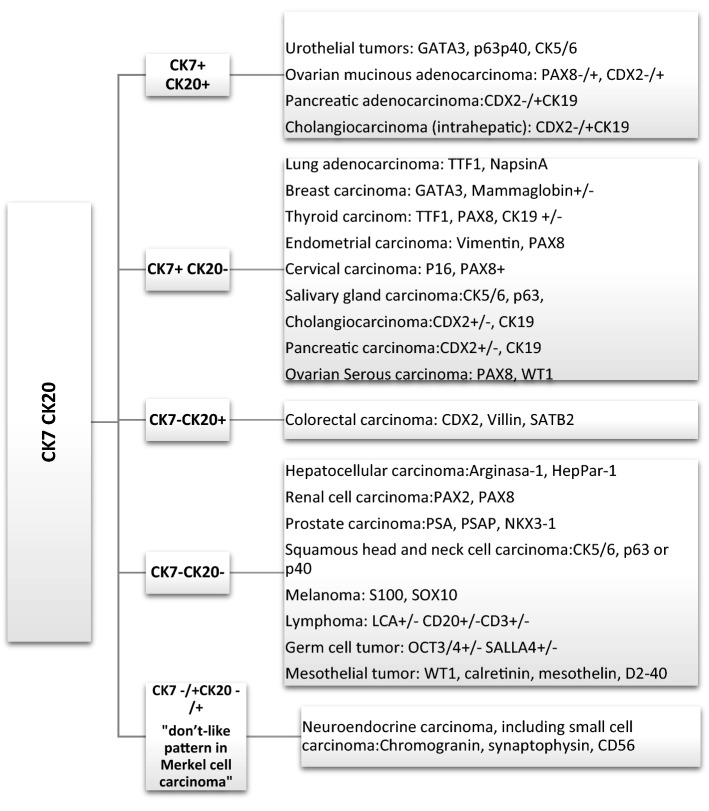
CK 7/CK 20 profile and additional markers. ( Adapted from J Gastrointestin Liver Dis, 2017 (26).1:69–79

The most common CK7 and CK20 profiles and other positive markers are shown in Fig. 1.

We propose a step-by-step algorithm to arrive at a CUP diagnosis (Table 3).

**Table 3:** step-by-step algorithm to arrive at a CUP diagnosis (adapted Rassy and Pavlidis, Nature Review 2020)^57^

Diagnosis	
Step one (Most likely cell lineage)	
CK + , S100 − , CD45 − , VIMENTIN ±	Carcinoma
CLA + , CD45 + , VIMENTIN + , CK − , S100 −	Lymphoma
S100 + , VIMENTIN + , CK − , CD45 −	Melanoma
S100 − ; VIMENTIN ± , CK − , *α*SMA,CD 45-	Sarcoma
Step two (types of carcinoma and categorizes into subgroups according to CK7/CK20 expression)	
CK7 and/or CK20; PAS	Adenocarcinoma
PLAP; OCT4; AFP; bHCG	Germ cell tumor
Chromogranin; synaptophysin; PGP9.5; CD56	Neuroendocrine carcinoma
CK5 or CK6; p63	SCC
Step three (categorizes carcinomas into subgroups according CK7/CK20 expression)	
CK7 + and CK20 +	Ovarian mucinous or pancreatic adenocarcinoma, urothelial carcinoma, cholangiocarcinoma
CK 7 − and CK 20 +	Colorectal or Merckel cell carcinoma
CK7 + and CK20 −	Lung adenocarcinoma, cholangiocarcinoma, breast, thyroid, endometrial, ovarian, cervical, salivary gland or pancreatic carcinoma
CK 7 − and CK 20 −	SCC, hepatocellular, renal cell, prostate, small-cell lung cancer, head and neck carcinoma
Step Four (origin)	
TTF1, Napsina A (CK 7 + /CK 20 −)	NSCLC (adenocarcinoma)
GCDFP-15; mammaglobin, GATA 3, ER (CK 7 + /CK 20 −)	Breast carcinoma
PSA, PAP, NKX 3,1 (CK 7 −/CK20 −)	Prostate carcinoma
CDX2, CEA, SATB2 (CK 7 −/CK20 +)	Colon carcinoma
ER, CA-125 (CK 7 + /CK 20 −)	Endometrial carcinoma
ER, CA-125, mesothelin, WT1 (CK7 + /CK 20 −)	Serous ovarian cancer
CA-125, S100 (CK7 + /CK 20 +)	pancreatic adenocarcinoma
CD 10, RCC (CK 7 −/CK20 −)	Renal cell carcinoma
TTF 1, thyroglobulin**,** PAX 8 (CK 7 + /CK 20 −)	Thyroid carcinoma
Hep Par-1, AFP, polyclonal CEA, CD 10, CD13, Arg 1 (CK 7 −/CK20 −)	Hepatocellular carcinoma

CK cytokeratin, *αSMA* α-smooth muscle actin, *AFP* α-fetoprotein, *CA-125* cancer antigen 125, *CDX2* caudal type homeobox 2, *CEA* carcinoembryonic antigen, *CLA* cutaneous lymphocyte-associated antigen, *ER* estrogen receptor, *GCDFP-15* gross cystic disease fluid protein 15, *HCG* human chorionic gonadotropin, *Hep Par-1* hepatocyte-specific antigen, *NKX 3,1* NK3 homeobox 1, *OCT4* octamer-binding transcription factor 4, *PAP* prostatic acid phosphatase, *PAS* periodic acid Schiff, *PAX 8* Paired box gene-8 protein, *PGP9.5* protein gene product 9.5, *PLAP* placental alkaline phosphatase, *PSA* prostate-specific antigen, *RCC* renal cell carcinoma marker, *SCC* squamous-cell carcinoma, *TTF1* thyroid transcription factor 1, *WT1* Wilms tumour protein, *NSCLC* non-small cell lung cancer

## Molecular diagnosis

There are basically two types of strategies when considering the use of a molecular study platform in patients diagnosed with CUP: diagnostic molecular platforms aimed at identifying the primary tumor and Sequencing platforms for tumor mutation profile characterization.

### Diagnostic molecular platforms aimed at identifying the primary tumor

These platforms base their results on the performance of a similarity score with the genetic or epigenetic characteristics of already known primary tumors. The procedure is based on the fact that, once the molecular profile of the tumor has been obtained, it is compared with the results of databases of cases of already known locations and histological types. The similarity of the molecular profile of the tumor evaluated with these patterns is assessed and a diagnosis is given that offers one (or several) locations, estimating the probabilities of each (similarity score).

There are several types of platforms with this approach: those that point to the genomic profile, which determines either the DNA gene expression or microRNA profile; and those that point to the epigenomic profile by characterizing the DNA methylation pattern of a certain number of genes associated with known tumors [17–21].The rate of concordance with respect to the diagnosis of occult primary tumor is around 82–97%.

Despite this, the impact on the clinical benefit of targeted therapy based on molecular studies remains controversial and the level of evidence and grade of recommendation is low, because they are based on short series or retrospective phase II studies [20, 22]. We have two prospective randomized studies completed in the last few years and neither demonstrate a significant benefit, except for the identification of clearly treatable primary tumors [23, 24]. (Level of evidence III, grade of recommendation B).

### Sequencing platforms for tumor mutation profile characterization

Sequencing with gene panels using Next Generation Sequencing (NGS) techniques makes it possible to identify mutations that, in a more or less significant percentage of cases, could be associated with a benefit from the use of drugs targeted at these same mutations.

The potential benefit of this approach based on the ultraselection of oncospecific treatments based on molecular profiling can identify actionable genomic alterations in 87% of patients, and in between 30–40% of these patients with clear treatment directed against the molecular target with ESCAT or OncoKB level of 2 or less [25–36] (Table 4). The main objection stems from whether the response potential of a drug to a given mutation is conditioned by the tumor type in which the mutation is found. That means the histological type context and tumor location may condition the response to the drug, and not just the mutational profile [31, 37–39].

**Table 4:** Potentially actionable genomic alterations

Identified actionable mutation	Westphalen et al. 2021 *n* = 346	Ross et al. 2020 *n* = 303	Ross et al. [27] *n* = 200	Löffler et al. [33] *n* = 55	Tothill et al. [25] *n* = 16	Gatalica et al. [26] *n* = 47	Krämer et al. [34] *n* = 4650
ALK, RET, ROS1, NTRK rearrangements	2%	1%	n.d	n.d	n.d	n.d	1%
PTCH1 (inactivating)	1%	1%	1%	n.d	13%	–	1%
SMO (activating)			1%	1%	1%	–	
AKT1	10%	8%	1%	5%	6%	2%	13%
PI3K			9%	4%	19%	8%	
BRCA1	6%	6%	2%	n.d	19%	–	6%
BRCA2			6%	n.d	6%	11%	
EGFR	2%	2%	6%	5%	6%	1%	2%
FGFR2	8%	n.d	n.d	n.d	n.d	n.d	n.d
MET	2%	n.d	n.d	n.d	n.d	n.d	n.d
BRAF V600	6%	3%	6%	5%	1%	3%	2%
ERBB2	6%	9%	8%	5%	1%	2%	7%
ERBB3	n.d		2%	n.d	1%	–	
TMB-high (≥ 16 mutation/MB)	6%	9%	n.d	n.d	n.d	n.d	10%
MSI-high	3%		n.d	n.d	n.d	n.d	

Identified actionable mutation	Targeted therapy	Ross et al. [27] *n* = *200*	Löffler et al. [33] *n* = *55*	Tothill et al. [25] *n* = *16*	Gatalica et al. [26] *n* = *47*	Krämer et al. [34] *n* = *4650* *Dataset foundation medicine*	Ross et al. 2020 *n* = 303	Gatalica et al. [54] *n* = 389	Varghese et al. [28] *n* = 150
ALK rearrangements	Alectinib	n.d	n.d	n.d	n.d	30/4650 (0.6%)	1%	–	1
RET rearrangements		n.d	n.d	n.d	n.d		1%	–	1
PTCH1 (inactivating)	Vismodegib	1/200 (1%)	n.d	2/16 (13%)	–	48/4650 (1.0%)	1%		2
SMO (activating)		1/200 (1%)	0/55 (0%)	0/16 (0%)	–				–
AKT1	Ipatasertib	2/100 (1%)	3/55 (5%)	1/16 (6%)	2%	608/4650 (13.1%)		–	3
PI3K		17/200 (9%)	2/55 (4%)	3/16 (19%)	8%		6%	8.5%	10
BRCA1	Olaparib	3/200 (2%)	n.d	3/16 (19%)	–	259/4650 (5.6%)	7%	1%	2
BRCA2		11/200 (6%)	n.d	1/16 (6%)	11%			1.3%	2
EGFR	Erlotinib + bevaciz umab	12/200 (6%)	3/55 (5%)	1/16 (6%)	1%	98/4650 (2.1%)	4%	–	–
FGFR		–	–	–	–	–	4%	–	3%
BRAF V600	Vemurafenib + cobimetinib	11/200 (6%)	3/55 (5%)	0/16 (0%)	3%	102/4650 (2.2%)	4%	4,2%	4%
ERBB2	Trastuzumab + per tuzumab + chemotherapy	16/200 (8%)	3/55 (5%)	0/16 (0%)	2%	329/4650 (7.1%)	7%	1.3%	5%
ERBB3		3/200 (2%)	n.d	0/16 (0%)	–			–	
TMB-High (≥ 16 mutations/MB)	Atezolizumab					438/4650 (9.4%)	9%	46/389(11.8%)	–
MSI-high	Pembrolizumab						1%	7/389 (1.8%)	–
PDL1 high expression	ICI therapy						14%	82/365 (22.5%)	–

Platforms that perform complete genomic profiling by massive genomic sequencing in the coding region of a predetermined number of cancer-related genes allow the detection of genomic alterations such as mutations, DNA insertions/deletions, copy number variations, and gene recombinations. They also allow the evaluation of repair genes (study of microsatellite instability) and the determination of the tumor mutational burden (TMB), which is of paramount importance for the indication of target treatments and/or immunotherapeutics [26, 27, 32].

There is currently consensus on the therapeutic agnostic indication for the use of NTRK fusion gene inhibitors or, in those tumors with a TMB with more than 10 mutations per megabase, for the use of immunotherapy with pembrolizumab [35, 40, 41]. (Level of evidence III, grade of recommendation B).

Finally, it should be noted that a major international umbrella trial is currently underway with the intention of demonstrating that ultraselection of treatments based on genomic profiling and target therapy may represent a new paradigm in the management of these patients even without remote knowledge of the most likely primary tumor [42].

## Recommendations for the use of molecular platforms

There is consensus among experts that the use of molecular platforms (MP) should be an ordinary part of the diagnostic process of CUP [25, 29, 30, 32, 33].

Patients who are candidates for MP should show an absence of clear clinical criteria for a known primary tumor, as well as an absence of clinical-immunohistochemical correlation. Initially, an anatomopathological morphological evaluation and immunohistochemical battery should be performed as precisely as possible, ensuring that after IHC, there is sufficient tissue to perform an MP analysis. If there are no clear results with initial IHC and a second and/or third IHC step, an MP should be used, not so much to identify the most likely primary tumor, but to characterize genomic alterations that may be associated with possible personalized target therapy treatments, especially in fit patients with a 0–1 ECOG and not very high LDH [8]. (Level of evidence III, grade of recommendation B).

## Oncospecific systemic treatment according to histopathologic and/or clinical criteria

There is a subgroup of patients, between 10–15% of all CUP, whose form of presentation and/or specific anatomopathological criteria are part of a favorable prognostic group, since they have a specific treatment equivalent to that of the known primary tumors which they resemble. Table 5 shows the different groups of CUP with specific treatment [6]. (Level of evidence IV, grade of recommendation B).

**Table 5:** Treatments for specific subsets of patients with CUP ( adapted from Hainsworth JD. www.uptodate.com)

Histopathologic subtype	Clinical features	Therapeutic approach
Adenocarcinoma	Women with isolated axillary adenopathy	= II breast cancer
	Women with peritoneal carcinomatosis	= Stage III ovarian cancer
	Men with elevated PSA or blastic bone metastases	= Advanced prostate cancer
	Colon cancer profile	= Advanced colon cancer
Adenocarcinoma or PDC	Single metastatic lesion	Definitive local therapy (resection and/or radiation therapy)
Squamous cell carcinoma	Cervical adenopathy	= head and neck cancer with involved neck nodes
	Inguinal adenopathy	Inguinal node dissection. Consider concurrent radiation therapy/chemotherapy (as in locally advanced cervical or anal cancer)
Poorly-differentiated carcinoma	Young men with midline tumor or elevated bHCG/AFP	= extragonadal germ cell tumor
Poorly-differentiated neuroendocrine carcinoma	Diverse clinical presentations	Treat with platinum/etoposide or paclitaxel/platinum/etoposide

*PDC* poorly differentiated carcinoma

However, most of these patients do not belong to any specific subtype or location, so the treatment of choice is empirical chemotherapy, with combinations containing a platinum agent plus another cytotoxic agent (taxanes, gemcitabine, irinotecan) [45], although this treatment provides poor results with low response rates (RR) of 15–20% and about nine months overall survival. [40, 46–53] Table 6. (Level of evidence II, grade of recommendation A).

**Table 6:** Prospective trials with chemotherapy regimens in patients with unfavorable CUP

Ref.	CT schedule	ORR	OS
Culine et al. [46]	Cisplatin + gemcitabine vs cisplatin + irinotecan	55 vs 38	8 vs 6
Greco et al. [47]	Cisplatin + docetaxel vs carboplatin + docetaxel	26 vs 22	8 vs 8
Huebner et al. [48]	Carboplatin + paclitaxel vs gemcitabine + vinorelbine	23.8 vs 20	11 vs 7
Dowel et al. [49]	Carboplatin + etoposide vs paclitaxel + 5-fluorouracil + leucovorin	19 vs 19	8.3 vs 6.4
Briasoulis et al. [50]	Irinotecan + oxaliplatin	13	2.7
Schuette et al. [51], Moller et al. [52], Hainsworth et al. [53]	Capecitabine + oxaliplatin	11.7–19	3.9 -9.7

It is important to point out that the IHC diagnosis is only indicative of the location of a primary tumor and should be considered by itself insufficient to guide treatment in that direction. Hence the concept of "suggestive primary cancer profile". This is where it makes sense to complement IHC information with NGS to ultra-detect mutations or other actionable genomic alterations. In these cases, a genomic platform could help us to identify a genomic profile and/or an actionable genomic alteration, or biomarkers of response to immunotherapy [27, 54]. (level of evidence III, grade of recommendation B).

## Surgical and radiotherapy treatment

When CUP are identified as localized disease, after complete staging (including PET) [55], local treatment can result in long disease-free intervals and improve the prognosis of these patients. The most common sites include liver, bone, lung, skin, adrenal gland, and lymph nodes.

An attempt should be made at treatment with surgery of the solitary lesion. If resection is not feasible, definitive local radiation therapy should be proposed. Also, consider radiation therapy for patients with risk factors for residual disease, for example, multiple involved nodes or extracapsular spread, to maximize the chance of local control.

The role of neoadjuvant or adjuvant chemotherapy is undefined. Empirical adjuvant chemotherapy is reasonable in this setting, particularly in patients with poorly differentiated carcinoma or if indicated in a predicted tumor. (Level of evidence IV, grade of recommendation B).

## Treatment based on suggesting primary cancer profile

The majority of patients with CUP (80–85%) are not included in the onco-specific treatment subgroups and their prognosis and treatment options are poor. Here arises the concept of “suggesting primary cancer profile” using the molecular cancer classifier (see Diagnostic molecular platforms aimed at identifying the primary tumor) to guide site-specific treatment [40].

Two retrospective non-randomized trials evaluating tumor type-specific therapies support this approach especially in patients with more responsive tumour types [20, 56]. However, two prospective studies (a Japanese phase II prospective and the European GEFCAPI-04 phase III trial) did not confirm the benefit in terms of progression-free survival or overall survival [23, 24].

So far, if primary site identification by these methods improves, outcomes remain to be seen, as these studies have numerous flaws such as non-optimal tumor type-specific therapy, cohorts enriched with resistant and unresponsive cancer types, or they are underpowered to detect survival benefit.

Otherwise, these molecular platforms can identify patients with tumors considered either sensitive or resistant to treatment. The benefits of this approach are most evident in patients predicted to have treatment-responsive tumor types [57]. Emerging non-randomized data favour the identification of certain subgroups of patients in whom a more specific treatment, similar to that for primary tumors which their conditions resemble, may be beneficial, such as renal-cell CUP, lung CUP and colorectal CUP. (Level of evidence III, grade of recommendation B).

## Personalized treatment according to actionable genomic alterations

Due to its poor prognosis and response to conventional platinum-based chemotherapy treatments, the discovery of potential therapeutic molecular targets is one of the challenges of CUP.

Retrospective studies have observed that most patients with CUP harbored ≥ 1 oncogenic driver mutation determined by NGS technique [27, 28] and in 65% of patients with CUP actionable mutations by liquid biopsy-based cell-free circulating-tumor DNA [29], suggesting the need for further investigation into the value of molecular profiling.

Actionable genomic alterations matched to targeted therapies (Table 7) have been studied mostly from case reports retrospectively in patients with CUP [26, 58–67] and also biomarkers of response to immunotherapy such as TMB-high and MSI-high, associated to an important clinical benefit in some cases.

**Table 7:** Targeted therapies in relation to the genomic alterations

Identified actionable genome alterations	Associated targeted therapies
ALK	Crizotinib, ceritinib, alectinib, brigatinib, lorlatinib
RET	Selpercatinib, pralsetinib
ROS1	Crizotinib, ceritinib, lorlatinib, repotrectinib
NTRK	Entrectinib, larotrectinib
PI3K	Temsirolimus, everolimus, alpelisib
BRCA1/2	Olaparib, niraparib, rucaparib, talazoparib
EGFR	Gefitinib, erlotinib, afatinib, dacomitinib, osimertinib
FGFR2/3	Pazopanib, ponatinib, erdafitinib
MET	Crizotinib, tepotinib, capmatinib
BRAF V600	Vemurafenib, encorafenib, dabrafenib, regorafenib
KRAS G12C	Sotorasib, adagrasib
ERBB2	Trastuzumab, lapatinib, pertuzumab, afatinib, neratinib, trastuzumab-deruxtecan, trastuzumab emtansina, tucatinib
SMO	Vismodegib
AKT1	Ipatasertib, capivasertib
IDH1	Ivosidenib
TMB-high	Immune checkpoint inhibitors
MSI-high	Immune checkpoint inhibitors

We have to take into consideration that some targeted therapies have already been approved by FDA for refractory and unresectable solid tumors such as larotrectinib and entrectinib (NTRK inhibitors) in NTRK fusion tumors with remarkable response [64] in CUP patients and pembrolizumab for MSI-high and TMB-high solid tumors [67]. (Level of evidence III, grade of recommendation B).

Apart from that there is a lack of prospective data from a large trial comparing platinum based chemotherapy to molecular alteration-based targeted therapy on this group, so nowadays the prognostic and predictive value of molecular profiling is pending and prospective studies examining biomarker-directed therapy on these cases (CUPISCO trial NCT03498521) [34] are ongoing.

## Conclusions

CUP represents 3–5% of all tumor diagnoses and is characterized by an aggressive clinical course, an unpredictable metastatic pattern, early dissemination, intrinsic resistance to treatment and poor prognosis. It is a biologically complex entity, with intrinsic genetic alterations, which condition a behavior different from that of the primary tumors which it most resembles.

Given the aggressiveness of most CUPs, it is important to make a diagnosis as early as possible, performing the diagnostic process in a standardized manner, both clinically and pathologically, reserving part of the tumor sample, if possible, to determine actionable mutations, especially in patients with ECOG 0–1.

The limitations facing the diagnosis and treatment of CUP remain a major challenge. Improvements in diagnostic techniques, including the latest generation of IHC markers and genomic molecular platforms, have helped to refine the detection of the possible primary in many cases.

New molecular platforms based on massive gene sequencing offer us a new horizon through the detection of actionable genomic alterations for which personalized targeted treatments are available. The combination of treatment according to the most likely primary tumor and appropriate treatment for actionable mutations will undoubtedly offer an enormous opportunity of benefit for many patients diagnosed with CUP in the future.
